# Multiple step saccades in simply reactive saccades could serve as a complementary biomarker for the early diagnosis of Parkinson’s disease

**DOI:** 10.3389/fnagi.2022.912967

**Published:** 2022-07-27

**Authors:** Wenbo Ma, Min Li, Junru Wu, Zhihao Zhang, Fangfang Jia, Mingsha Zhang, Hagai Bergman, Xuemei Li, Zhipei Ling, Xin Xu

**Affiliations:** ^1^State Key Laboratory of Cognitive Neuroscience and Learning and IDG/McGovern Institute for Brain Research, Division of Psychology, Beijing Normal University, Beijing, China; ^2^Edmond and Lily Safra Center for Brain Sciences, The Hebrew University of Jerusalem, Jerusalem, Israel; ^3^Department of Cadre Medical Service, The First Clinical Center, Chinese PLA General Hospital, Beijing, China; ^4^Senior Department of Neurosurgery, Chinese PLA General Hospital, Beijing, China

**Keywords:** saccade, corrective saccades, MPTP, Parkinson’s disease, diagnosis

## Abstract

**Objective:**

It has been argued that the incidence of multiple step saccades (MSS) in voluntary saccades could serve as a complementary biomarker for diagnosing Parkinson’s disease (PD). However, voluntary saccadic tasks are usually difficult for elderly subjects to complete. Therefore, task difficulties restrict the application of MSS measurements for the diagnosis of PD. The primary objective of the present study is to assess whether the incidence of MSS in simply reactive saccades could serve as a complementary biomarker for the early diagnosis of PD.

**Materials and methods:**

There were four groups of human subjects: PD patients, mild cognitive impairment (MCI) patients, elderly healthy controls (EHCs), and young healthy controls (YHCs). There were four monkeys with subclinical hemi-PD induced by injection of 1-methyl-4-phenyl-1,2,3,6-tetrahydropyridine (MPTP) through the unilateral internal carotid artery and three healthy control monkeys. The behavioral task was a visually guided reactive saccade.

**Results:**

In a human study, the incidence of MSS was significantly higher in PD than in YHC, EHC, and MCI groups. In addition, receiver operating characteristic (ROC) analysis could discriminate PD from the EHC and MCI groups, with areas under the ROC curve (AUCs) of 0.76 and 0.69, respectively. In a monkey study, while typical PD symptoms were absent, subclinical hemi-PD monkeys showed a significantly higher incidence of MSS than control monkeys when the dose of MPTP was greater than 0.4 mg/kg.

**Conclusion:**

The incidence of MSS in simply reactive saccades could be a complementary biomarker for the early diagnosis of PD.

## Introduction

Saccades are rapid eye movements that redirect the fovea from one object of interest to another. A typical saccade consists of a primary saccade that covers all or most of the distance between the fixation point and the target location, which might be followed shortly by a small amplitude saccade (corrective/secondary saccade) if required. Corrective saccades (CS) have been frequently observed in children and young and elderly healthy subjects (Cohen and Ross, 1978; White et al., 1983). Thus, it has been well accepted that CS is a physiological behavior (Troost et al., 1974).

However, eyes do not always jump with the typical form and sometimes they engage in a series of at least two smaller amplitude (hypometric) saccades–namely, multiple step saccades (MSS) (Troost et al., 1974). Although MSS is occasionally observed in healthy subjects (Kimmig et al., 2002; Van Donkelaar et al., 2007), it is clearly more pronounced in Parkinson’s disease (PD) patients (Jones and DeJong, 1971; Corin et al., 1972; Troost et al., 1974; Teräväinen and Calne, 1980; White et al., 1983; Hotson et al., 1986; Lueck et al., 1990, 1992; Van Gisbergen et al., 1992; Kimmig et al., 2002) and non-human primates with dopamine depletion (PD monkeys) in the basal ganglia (Brooks et al., 1986; Kato et al., 1995). Thus, MSS is assumed to be a non-physiological behavior (Troost et al., 1974).

A consistent finding among previous studies is that the incidence of MSS in PD patients is significantly higher than that in elderly healthy controls (EHCs) during voluntary saccades such as memory guided saccades (Teräväinen and Calne, 1980; Crawford et al., 1989; Lueck et al., 1992; Van Gisbergen et al., 1992; Kimmig et al., 2002; Ying et al., 2008; Blekher et al., 2009). Furthermore, it has been argued that the incidence of MSS in memory guided sequential saccades could serve as a biomarker for the diagnosis of PD (Blekher et al., 2009). However, practically, memory guided sequential saccade tasks are usually difficult for elderly subjects to perform, particularly for neurodegenerative patients, because participants need to inhibit the reactive saccades to the onset of visual stimulus and then generate saccades based on their memory (Gaymard et al., 1998). Previous studies have found that PD patients made significantly more errors in memory guided saccade tasks (Crawford et al., 1989; Van Gisbergen et al., 1992). Such task difficulties restrict the clinical application of measuring MSS in the diagnosis of PD. Thus, a critical question is whether the incidence of MSS in simple saccade tasks, such as reactive saccades, could provide useful information for the diagnosis of PD.

While the reported incidences of MSS are consistent for memory guided saccades among previous studies, the results are inconsistent for reactive saccades. Some studies reported that, compared with elderly healthy subjects, the incidences of MSS are significantly higher in both PD patients (Jones and DeJong, 1971; Corin et al., 1972; White et al., 1983) and PD monkeys (Brooks et al., 1986; Tereshchenko et al., 2015), but others reported no significant difference (Crawford et al., 1989; Lueck et al., 1990, 1992; Van Gisbergen et al., 1992; Kimmig et al., 2002; Blekher et al., 2009). We think that a possible reason for such inconsistency might be the different definitions of MSS. Some previous studies excluded CS from the analysis of MSS (Troost et al., 1974; Bötzel et al., 1993; Van Donkelaar et al., 2007), while others considered CS as a part of MSS (Becker and Fuchs, 1969; Oliva, 2001). To make a comparison with previous studies, we firstly analyzed the incidence of mixed MSS and CS, and then dissociate CS from MSS for data analysis.

It has been noticed that the impairments of vertical saccades are more severe than horizontal saccades in PD patients (Lemos et al., 2016; Jung and Kim, 2019). One study argued that the characteristics of vertical saccades could serve as a complementary biomarker for the diagnosis of PD (Waldthaler et al., 2019). To the best of our knowledge, no study has compared the incidence of MSS between vertical and horizontal saccades. Therefore, one of the objectives of the present study was to address whether the incidence difference of MSS between vertical and horizontal saccades could also serve as a complementary biomarker for PD diagnosis.

To assess the usefulness of MSS in reactive saccades for the diagnosis of early PD, we studied the incidence of MSS in 1-methyl-4-phenyl-1,2,3,6-tetrahydropyridine (MPTP)-induced subclinical hemi-PD monkeys, because it is rare to see early PD patients in the hospital where we collected data of PD patients. In addition, since MPTP selectively damages the dopaminergic neurons in the substantia nigra pars compacta (Langston, 2017), the function of dopaminergic circuit in basal ganglia is impaired after MPTP injection (Israel and Bergman, 2008). Moreover, the substantia nigra pars reticulata directly sent output to the intermedia and deep layers of superior colliculus–a saccadic center in brain (Hikosaka et al., 2014), the superior colliculus is dysfunctional in PD monkeys (Rolland et al., 2013). Therefore, this study could help to understand the role of basal ganglia-superior colliculus circuit in the development of MSS.

Furthermore, to the best of our knowledge, previous studies have only compared the incidence of MSS between PD patients and EHC, but no one has investigated whether there is a difference in MSS between PD and mild cognitive impairment (MCI) patients. Thus, the specificity of MSS for the diagnosis of PD is unclear. To address this knowledge gap, we compared the incidence of MSS between PD and MCI patients. We set MCI patients as a control in the present study for the following consideration. PD and MCI are two common neurodegenerative diseases and share certain pathological changes (Xu et al., 2012; Tosto et al., 2015).

## Materials and methods

### Participants in the human study

We recruited four groups of participants in the present study. These four groups included PD patients (*n* = 37), MCI patients (*n* = 37), age-matched EHCs (*n* = 37), and young healthy controls (YHCs) (*n* = 37). The demographic data and clinical scores were shown in Table 1. All participants had normal or corrected-to-normal vision. All participants have written informed consents to take part in the study. The experimental protocols were approved by the Ethics Committee of Beijing Normal University and the Chinese PLA General Hospital (Medical School of Chinese PLA).

**TABLE 1:** Demographic and clinical characteristics of the human subjects.

	YHC	EHC	MCI	PD
*N* (male/female)	37 (20/17)	37 (27/10)	37 (28/9)	37 (24/13)
Age in years^a^	22.68 ± 2.92	62.76 ± 7.13	75.24 ± 8.88	62.49 ± 6.97
MMSE^a^	29.49 ± 0.64	25.77 ± 4.31	23.38 ± 3.94	25.95 ± 3.73
MoCA^a^	29.52 ± 0.61	–	23.38 ± 3.94	21.95 ± 4.83
MDS-UPDRS^a^	–	–	–	56.81 ± 13.41^b^
H-Y scale^a^	–	–	–	2.62 ± 0.39^b^

^a^Mean ± SD.

^b^MDS-UPDRS III and H-Y scale were evaluated only for PD patients. The table shows the scores in medicine off state.

All participants except the EHC completed the Folstein mini-mental state examination (MMSE) and Montreal Cognitive Assessment (MoCA) for cognitive function evaluation. Considering the fact that it is rare to see the real healthy participants in hospital, we recruited young and elderly healthy participants in the college community and the residential community, respectively. To make the present study more practical, data collection was carried out by different experimenters in different places. For PD and MCI patients, their cognitive function was evaluated by neurologists in hospital. For YHCs, their cognitive function was evaluated by using MMSE and MoCA tests in university. For EHCs, their cognitive function was evaluated by using MMSE in residential community. For PD patients, MMSE, and MoCA were performed after medication on-state (approximately 1 h after taking levodopa and/or amantadine); the Part 3 of the Movement Disorders Society-revised Unified Parkinson’s Disease Rating Scale (MDS-UPDRS-motor scores), Hoehn and Yahr scale (H&Y stage) were administered during the medication off-state (approximately 4 h after taking the medicine). The UPDRS was administered only to PD patients. The data of saccadic eye movements were collected from PD patients during the medication off-state.

Mild cognitive impairment patients meet the diagnosis criteria of MCI according to the National Institute on Aging Alzheimer’s Association workgroups in 2011 (Albert et al., 2011). The main diagnostic criteria were as follows: (1) Concern regarding a change in cognition; (2) Impairment in one or more cognitive domains by cognitive assessment (MMSE and MoCA tests); (3) Preservation of independence in functional abilities; (4) Not demented. MCI patients continued their regular medication routine.

### Participants in the non-human primate study

Seven male rhesus monkeys were recruited in the present study, including four subclinical hemi-PD (9–12 kg, 12–14 years old) and three healthy control monkeys (10–12 kg, 12–14 years old). Four subclinical (prodromal) hemi-PD monkeys were modeled by injection of MPTP (dissolved in saline with concentration of 0.12 mg/ml) through the unilateral internal carotid artery (Bankiewicz et al., 1986) by a peristaltic pump (RWD Life Science Co., Ltd., Shenzhen, China) with flow rate 1.54 ml/min. The doses of MPTP injection were referred to the previous study (Ovadia et al., 1995) with 0.38, 0.40, 0.41, and 0.43 mg/kg for the four hemi-PD monkeys, respectively. All monkeys were housed in separate cages with a 12 h light/dark cycle. Before training, each monkey was surgically implanted with a head post and two eye coils. The experimental protocols and surgical procedures were approved by the Ethics Committee of Beijing Normal University.

### Experimental task

We used a visually guided reactive saccade task to study MSS. We collected eye movement data from one block of trials for each participant.

The task in the human experiment consisted of 40 or 60 trials in a block according to individual participant’s affordability. Since some PD and MCI patients had difficulty performing 60 trials in a block we reduced the number of trials to 40 for PD and MCI patients, while for YHCs and EHCs the number of trials in a block was 60. To balance the trial numbers among different groups, we randomly picked up 40 trials from each block of healthy controls for further data analysis.

As for monkey studies, the trial number was varied from 100 trials to 800 trials among blocks. To balance the trial numbers among blocks, we randomly picked up 100 trials form blocks with trial numbers larger than 100. It cost about 5 min for monkeys to perform 100 trials in a block.

**FIGURE 1:**
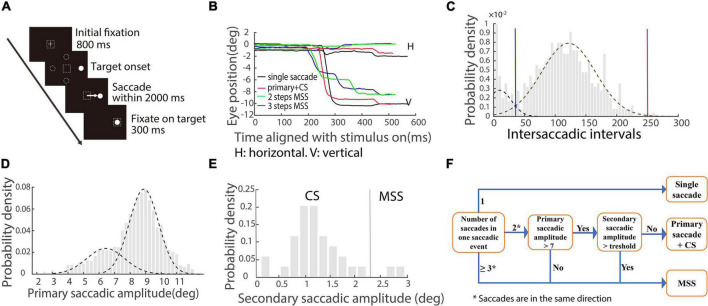
Schematic illustration of saccadic tasks and methods of quantifying saccades. **(A)** The paradigm of the visually guided reactive saccade task: White cross and circle represent fixation point and target, respectively. Dashed squares indicate the location of fixation. Dashed circles indicate other potential locations of the target. The white arrow represents the required saccade. **(B)** Exemplified eye trace of a PD patient. The *X*-axis represents the time aligned with the saccadic target onset. The *Y*-axis represents the eye position. Different colors denote different types of saccadic events. **(C)** The distribution of intersaccadic intervals. Dotted lines represent two unimodal distributions. The blue line is the cross point of the two unimodal fitting curves. The red line is the right zero point of the right unimodal fitting curve. **(D)** The distribution of primary/largest saccadic amplitudes. Two dotted lines indicate two unimodal distributions. **(E)** The distribution of secondary saccadic amplitude in which the first responsive saccadic amplitude is ≥7°. The vertical line indicates the mean + 2 × STD of the distribution. **(F)** Illustration of classifying different types of saccadic events.

Visually guided reactive saccade task (Figure 1A). Each trial began with a white cross (fixation point) appearing at the center of the screen for 800 ms. Simultaneously, with the disappearance of the fixation point, a white dot (target) appeared in one of four peripheral locations randomly (right, left, up, and down, with eccentricity of 10°). The size of the fixation points and target were 1° in length or diameter, respectively. Participants were instructed to fixate at the central cross (check window 4° in radius) and then make a saccade toward the target as accurately and fast as possible. The target disappeared after the eye entered and was maintained in the check window (4° in radius) for 300 ms. A blank screen was interposed between trials with an interval of 800 ms. It only took 3–4 s per trial, so each participant in the human study spent approximately 4 min on this test.

### Data acquisition

In human experiments, eye movements were monitored at 1 kHz with a head-restrained infrared video-based eye tracker (Eye Link 1000 desktop mount, SR Research Ltd., Kanata, ON, Canada; EM-2000R, Jasmine Science and Technology Ltd., Beijing, China). Participants were seated in a dark room 57 cm away from the monitor (XL2720-B; resolution: 1920 × 1080; 27-inch; refresh rate: 100 Hz). The system was calibrated prior to the experiment by having the participants make saccades to nine targets forming a rectangle (3 × 3 targets). The online check window (maximum calibration error) of the eye tracker is 2° in radius. The average calibration error was 1.65°. The background luminance of the monitor was 0.08 cd/m^2^, and the luminance of visual stimuli was 23.9 cd/m^2^. Stimuli presentation and behavioral data collection were controlled by MATLAB (R2009b; MathWorks, Natick, MA, United States) with Psychtoolbox (PTB-3) running on a Windows system PC (HP).

For the monkey experiment, eye position signals were recorded using the scleral eye coil technique (Crist Instrument Company, Hagerstown, MD, United States), and data were sampled at 1 kHz. Visual stimuli were displayed on a 27-inch screen (XL2720-B, resolution: 1920 × 1080, refresh rate: 144 Hz) that was placed 57 cm in front of the monkeys’ eyes. The background luminance of the monitor was 0.25 cd/m^2^, and the luminance of the visual stimuli was 341 cd/m^2^. We used a Windows PC system (DELL) to control the visual display and to run the real-time data acquisition system (Monkey Logic; NIH, Bethesda, MD, United States). The calibration procedure of the monkeys was similar to that in the human experiments. The recorded eye movement data were analyzed offline in MATLAB (R2017b; MathWorks, Natick, MA, United States).

Trials in which participants blinked after the target onset were excluded from further analysis. The average number of analyzable trials per participant group were 37, 36, 34, and 32 for YHC, EHC, MCI, and PD groups, respectively. Error trials, including fixation breaks and incorrect directions, were also excluded from further analysis. Overall, the excluded percentage of trials in the human study was 3.1, 6.7, 8.4, and 14.7% in the YHC, EHC, MCI, and PD groups, respectively. The excluded percentage of trials in the monkey studies was 3, 12, 17, and 26% for four hemi-PD monkeys and 1% for three control monkeys.

### Quantitative measures of saccades

A velocity threshold was set to find all responsive saccades from target onset to the end of the trial. The velocity threshold is the mean velocity ± 2.58 × STD (99% confidence interval) during a time interval of 200 ms prior to the target onset. The first responsive saccade was defined as a saccade with a minimum amplitude of 2° and a minimum latency of 30 ms, and its direction was toward the target location. While we plotted the eye traces, we found that there were different types of saccades with varied spatiotemporal properties (Figure 1B). To well classify the different types of saccades, we combined all responsive saccades within a trial together as one saccadic event if the intersaccadic intervals between adjacent saccades were within the two boundaries (blue and red vertical lines) of the distribution, as shown in Figure 1C (an example of the EHC group). We made the two unimodal fitting curves by employing maximum likelihood estimation. The blue line is the cross point of the two unimodal fitting curves. The red line is the right zero point of the right unimodal fitting curve. Since the distributions of intersaccadic intervals are different among the four groups of participants, the boundaries are varied.

From the exemplified eye traces shown in Figure 1B, it is obvious that there are different types of saccadic events, including typical saccades (a single saccade and a single saccade followed by a CS, black and red traces) and MSS with 2 or 3 steps (green and blue traces). The amplitude distribution of primary/largest saccades contains two separated unimodal distributions (Figure 1D, data of EHC), which supports at the population level that there are two different types of saccades, i.e., typical saccades and MSS. We will classify CS (a possible component of typical saccades) and MSS by the following criteria for further data analysis.

We first classified CS based on previous findings (Cohen and Ross, 1978). It has been reported that for a 10° required saccade, if the amplitude of the primary responsive saccade is ≥7°, the probability of generating CS is high (Cohen and Ross, 1978). Thus, we analyzed our data about the distribution of the amplitudes of secondary saccades for each group of participants (Figure 1E, EHC data), while the amplitudes of the first responsive saccades were ≥7°. CS is defined as the amplitude of secondary saccade in a saccadic event being less than a threshold, i.e., the mean amplitude + 2 × STD of secondary saccades and the direction is toward the target location.

We then classified MSS if a saccadic event met any one of the following criteria: (1) The number of saccades within a saccadic event is ≥3; (2) The number of saccades within a saccadic event is two, and the amplitude of the first responsive saccade is <7°; (3) The number of saccades within a saccadic event is two, the amplitude of the first responsive saccade is ≥7°, and the amplitude of the secondary saccade is ≥ the threshold. The directions of all mentioned saccades are the same. To help understand the logic and process of classification of the saccadic events, we schematically summarized the aforementioned definitions in Figure 1F.

To ensure that there was a sufficient number of correct trials for data analysis, the incidence of MSS was calculated when the correct rate of a session was ≥70%. In addition, the incidence of MSS in horizontal and vertical saccades was calculated when the correct rates of the two directions were ≥ the mean − 1.5 × STD (minimal trial number was 10) of each group of participants.

### Statistical analysis

The Kruskal–Wallis test (a non-parametric approach to one-way ANOVA) was applied to determine the significant difference among four independent groups of participants based on the incidence of MSS and CS. This was corrected by the Bonferroni correction. If there were significant differences among the four groups of participants, a *post-hoc* test was performed to determine the significance between each pair of participants either by the Wilcoxon rank-sum for unpaired tests or by the Wilcoxon signed-rank for the paired test. The alpha level was set to 0.05.

Furthermore, we employed a curve fitting tool (MATLAB, cftool function) to examine the relationship between MSS/CS and scores of UPDRS motor, MMSE and MoCA in PD group. We justified the goodness of fit curves based on the statistical results of the fitting function, including the sum of squares due to error (SSE), the root mean squared error (RMSE), the coefficient of determination (R-square), and the degrees-of-freedom adjusted coefficient of determination (adjusted R-square).

According to our results, we considered two useful parameters, i.e., the incidence of MSS and the incidence difference of MSS between vertical and horizontal saccades, that might help discriminate PD from the EHC or MCI group. We first applied a logistic regression model to predict the probability of PD by using the two parameters. The formula of this model is logit(*P*_*PD*_) = *a*_0_ + *a*_1_*x*_1_ + *a*_2_*x*_2_ + ε, where *x*_*1*_ is the incidence of MSS, *x*_*2*_ is the incidence difference of MSS between the vertical and horizontal saccades, *a*_*0*_ is a constant, *a*_*1*_ is the coefficient of *x*_*1*_, *a*_*2*_ is the coefficient of *x*_*2*_, ε is the random error, and *P*_*PD*_ is the probability of being diagnosed with PD. *a*_*0*_, *a*_*1*_, *a*_*2*_, and ε were determined by maximum likelihood estimation. We set the alpha level of the regression model to be 0.05. We next obtained the distribution of *P*_*PD*_ for each group of participants. Finally, we used *P*_*PD*_ to plot the receiver operating characteristic (ROC) curve and calculated the area under the curve (AUC), which indicates the ability to discriminate PD from the EHC and MCI groups by using the logistic regression model. We justify the predictors by the AUC which is the output of the logistic regression model.

## Results

### Results of the human experiment

#### Incidence of multiple step saccades and corrective saccades

**FIGURE 2:**
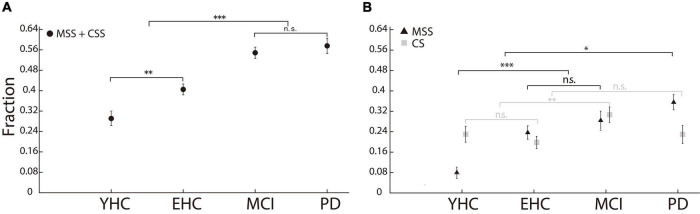
Incidence of MSS and CS in the human study. **(A)** The incidence of MSS including CS. There was no significant difference in MSS incidence between PD and MCI. **(B)** The incidence of MSS and CS, respectively. The incidence of MSS in PD was significantly higher than that in YHC, EHC, and MCI participants. Error bars show the standard error of the mean; *p < 0.05, ^**^p < 0.01, ^***^p < 0.001, n.s., no significant difference (Wilcoxon rank-sum test).

We first compared the incidence of MSS among four groups of human participants. If the CS was not excluded from MSS analysis, the incidence of multiple saccades in PD was significantly higher than that in both the EHC and YHC groups (*p* < 0.001, Wilcoxon rank-sum test, Figure 2A), which is consistent with some previous findings (Jones and DeJong, 1971; Corin et al., 1972; White et al., 1983). However, while MCI patients also showed significant differences comparing to EHC and YHC (*p* < 0.001, Wilcoxon rank-sum test), there was no significant difference between the PD and MCI patients (*p* > 0.05, Wilcoxon rank-sum test). Considering that MSS and CS might reflect non-physiological and physiological behaviors (Troost et al., 1974), we dissociated CS from MSS and compared their incidence among the four groups of participants, respectively (Figure 2B). First, the incidence of MSS in PD was significantly higher than that in YHC, EHC, and MCI groups (*p* < 0.05 for all comparisons); the incidences of MSS in the MCI and EHC groups were not significantly different (*p* > 0.05), while the incidences of MSS in the MCI and EHC groups were higher than those in the YHC group (*p* < 0.001 for all comparisons). Second, regarding the incidence of CS, only the MCI group showed a higher incidence of CS than the EHC and YHC groups (*p* < 0.05), whereas there was no significant difference among the EHC, YHC, and PD groups (*p* > 0.05, Kruskal–Wallis test, Bonferroni correction, *alpha* = 0.05/6).

#### Correlation between incidence of multiple step saccades/corrective saccades and scores of unified Parkinson’s disease rating scale motor, mini-mental state examination, and Montreal Cognitive Assessment, respectively

**FIGURE 3:**
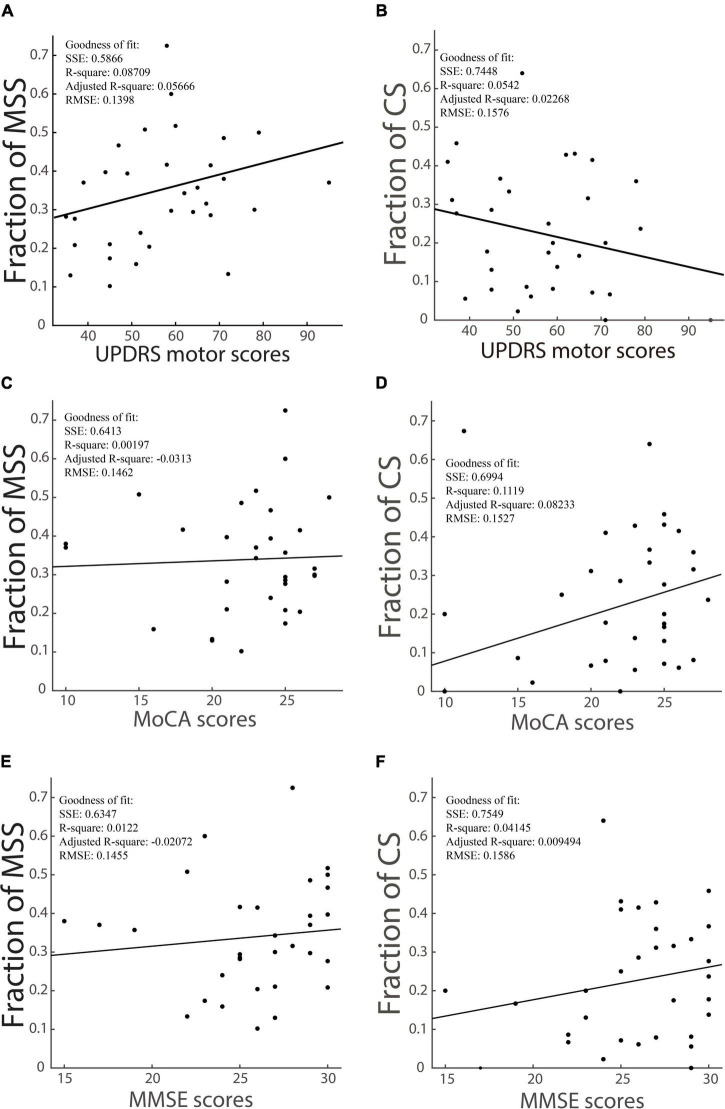
The correlation between the incidence of MSS, the incidence of CS and the UPDRS motor scores, scores of cognitive tests of PD patients. The incidence of MSS is modestly and positively correlated with UPDRS motor scores, as shown in panel **(A)**, while the incidence of CS has a slightly negative correlation with UPDRS motor scores, as shown in panel **(B)**. The incidence of MSS is not correlated with MoCA **(C)** and MMSE **(E)** scores, while the incidence of CS has a modestly positive correlation with MoCA **(D)** and MMSE **(F)** scores. SSE, the sum of squares due to error; RMSE, the root mean squared error; R-square, the coefficient of determination; Adjusted R-square, the degrees-of-freedom adjusted coefficient of determination.

We have shown that the incidence of MSS in the PD group was significantly higher than that in YHC, EHC, and MCI groups, whereas the incidence of CS was not significantly different between the PD group and YHC, EHC, and MCI groups. Here, we hypothesized that the incidence of MSS rather than CS might increase as the motor deficits of PD patients become more severe. To test this hypothesis, we analyzed the correlation between UPDRS motor scores and the incidence of MSS and CS. After data selection by the correct rate >70%, the number of PD patients in this analysis was 32. While the incidence of MSS and UPDRS motor scores showed a modestly positive correlation (R-square: 0.087) (Figure 3A), the incidence of CS and UPDRS motor scores showed no significant correlation (R-square: 0.023) (Figure 3B). Moreover, to test the relationship between MSS/CS and the scores of MMSE and MoCA, we did the same correlation analysis. The results showed that the incidence of MSS was not significantly correlated with the scores of MMSE and MoCA (R-squares: 0.0019 and 0.012) (Figures 3C,E), whereas the incidence of CS showed a modestly positive correlation with the scores of MMSE and MoCA, respectively (R-squares: 0.11 and 0.041) (Figures 3D,F). Such results support the argument that MSS might be a pathological behavior whereas CS be physiological behavior.

#### Incidence of multiple step saccades in horizontal and vertical saccades

**FIGURE 4:**
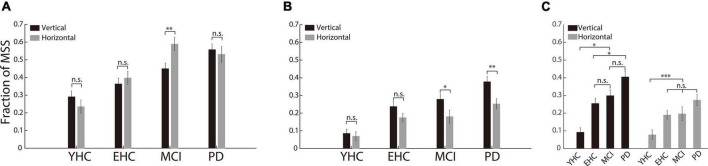
The incidence of MSS in horizontal and vertical saccades. **(A)** The incidence of MSS combined with CS between horizontal and vertical saccades. There was no significant difference in MSS incidence between vertical and horizontal saccades in any of the four groups of participants. **(B)** The incidence of MSS between the horizontal and vertical saccades. There was a significant difference between the vertical and horizontal saccades in PD and MCI patients. **(C)** The comparison of the MSS incidence among the four groups of participants. The incidence of MSS in vertical saccades was significantly higher in PD than in YHC, EHC, and MCI participants. Error bars show the standard error of the mean; *p < 0.05, **p < 0.01, ***p < 0.001, n.s., no significant difference (Wilcoxon rank-sum and sign-rank tests).

While CS was not excluded from MSS analysis, the incidences of MSS were not significantly different between vertical and horizontal saccades in YHC, EHC and PD groups (*p* > 0.05, Figure 4A). In MCI group, the incidence of MSS in horizontal saccades was higher than in vertical saccades (*p* < 0.01, Figure 4A). However, while CS was excluded from MSS analysis, the incidence of MSS was significantly higher in the vertical saccade group than in the horizontal saccade group in the PD and MCI groups (*p* < 0.01, *p* < 0.05 for the PD and MCI groups, respectively, Figure 4B). Moreover, while the incidence of MSS in vertical saccades was the highest in the PD group (*p* < 0.05 for the comparisons of PD vs. EHC and PD vs. YHC, respectively Figure 4C), the incidence of MSS in horizontal saccades was not significantly different both between the PD and EHC groups and between the PD and MCI groups (*p* > 0.05, Figure 4C). Such results indicate that the incidence of MSS in vertical saccades provides more reliable information than in horizontal saccades for the diagnosis of PD.

#### Logistic regression model and receiver operating characteristic analysis for discriminating Parkinson’s disease from elderly healthy control and mild cognitive impairment groups

**FIGURE 5:**
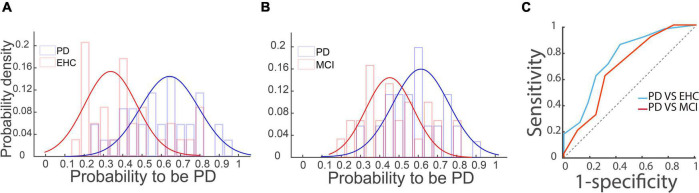
Logistic regression and ROC analysis of the PD vs. EHC and PD vs. MCI groups. **(A,B)** The distributions of the probability of PD based on the logistic regression analysis are shown in panels **(A,B)** for the PD vs. EHC and PD vs. MCI groups, respectively. Blue and red curves represent the two unimodal distributions of the PD and EHC/MCI groups, respectively. **(C)** ROC curve of the probability of PD between the PD and EHC and MCI groups. The *X*-axis represents 1-specificity, and the *Y*-axis represents sensitivity. The AUCs of PD vs. EHC (blue curve) and PD vs. MCI (red curve) were 0.76 and 0.69, respectively. The dotted line is the diagonal line.

Thus far, our results have shown that the incidence of MSS in the PD group is significantly higher than that in YHC, EHC, and MCI groups, and it occurs more frequently in vertical saccades than in horizontal saccades. To test the likelihood of discriminating PD from the EHC and MCI groups, we first performed logistic regression analysis (detailed information given in Section “Statistical analysis”). The distributions of the probability of being PD are shown in Figures 5A,B for PD vs. EHC and PD vs. MCI, respectively. It is obvious that the distributions between the PD group and EHC, MCI groups were different. To further measure the ability to discriminate PD from EHC and MCI by employing the probability of PD, we then plotted the ROC curve and obtained AUCs of 0.76 and 0.69 for the PD vs. EHC and PD vs. MCI groups, respectively (Figure 5C). Such results indicate that the incidence of MSS in reactive saccades could be a complementary biomarker for the diagnosis of PD.

### Results of the monkey experiment

To investigate whether the incidence of MSS could serve as a complementary biomarker for the diagnosis of PD in the early stage, we compared the incidence of MSS between four subclinical hemi-PD monkeys and three healthy monkeys. Despite the fact that these four monkeys did not show any typical PD motor symptoms, we are able to confirm the effect of MPTP injection by the following observations. Firstly, immediately after MPTP injection, the pupil size of ipsilateral injection side became significantly smaller comparing to the contralateral side which is consistent to the previous report (Metzger and Emborg, 2019). However, about 30 min later, the pupil size reversed between ipsilateral and contralateral sides. Secondly, as soon as the start of MPTP injection, the rate of heartbeat increased about 10–20%. Thirdly, after MPTP injection, monkeys lost appetite and reduced weight about 10–20%. Since the stage of subclinical PD is prior to the early stage of PD regarding the natural progress of PD and considering the fact that the severity of behavioral impairments (symptoms) increases following the development of PD, thus, the phenomenon of increased MSS in subclinical PD will also be observed in the early stage of PD. Before MPTP injection, two hemi-PD monkeys were trained to do some saccadic tasks, whereas other two monkeys were naïve to the saccadic tasks.

### Incidence of multiple step saccades in the monkeys

**FIGURE 6:**
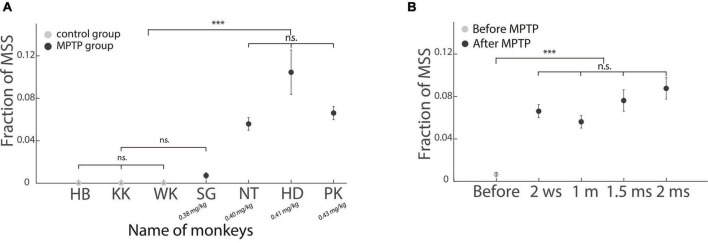
Incidence of MSS in healthy and subclinical hemi-PD monkeys. **(A)** Comparing the incidence of MSS between healthy and subclinical hemi-PD monkeys. The incidence of MSS was significantly higher in the three hemi-PD monkeys with MPTP dose larger than 0.4 mg/kg. Please be aware that the dosage of MPTP increases from left to right. **(B)** The incidence of MSS before and after MPTP injection in one hemi-PD monkey (PK). The *X*-axis is the time (w: week, m: month). The incidence of MSS increased significantly after MPTP injection. Error bars show the standard error of the mean; ^***^p < 0.0001, n.s., no significant difference (Wilcoxon rank-sum test).

As shown in Figure 6A, when the injection dose of MPTP was ≥0.4 mg/kg, three subclinical hemi-PD monkeys showed a significantly higher incidence of MSS than three control monkeys and one hemi-PD monkey SG (*p* < 0.001, Wilcoxon signed-rank test). We next compared the incidence of MSS before and after the MPTP injection in monkey PK. It is clear that the incidence of MSS significantly increased after MPTP injection (Figure 6B).

**FIGURE 7:**
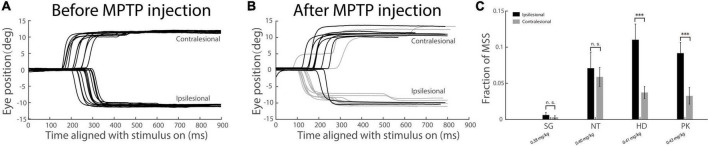
Comparison of the incidence of MSS between ipsilesional and contralesional saccades in subclinical hemi-PD monkeys. **(A,B)** Exemplified horizontal eye traces of monkey PK before and after MPTP injection. The *X*-axis represents the time aligned with the saccadic target onset. The *Y*-axis represents the eye position. Black traces denote one step saccades, or one primary saccade followed by a corrective saccade. Gray traces denote MSSs. The incidence of MSS is greater in ipsilesional saccades than that in contralesional saccades. **(C)** The incidence of MSS in population level between the ipsilesional and contralesional saccades. There is a significant difference between the ipsilesional and contralesional saccades in monkey HD and PK who received bigger dose of MPTP injection. Error bars show the standard error of the mean; ^***^p < 0.001, n.s., no significant difference (Wilcoxon rank-sum and sign-rank tests).

Since our PD monkeys were induced by unilateral injection of MPTP, it is interesting to see whether the incidence of MSS is different between ipsilesional and contralesional saccades. To make it visible, we randomly picked up 10 trials from left and right saccades, respectively, in two exemplified sessions one before and one after MPTP injection. The horizontal eye positions were plotted in Figures 7A,B. It clearly showed that the number of trials with MSS (gray traces) increased after MPTP injection. Moreover, the incidence of MSS in ipsilesional saccades was higher than that in contralesional saccades (Figure 7B). The population results showed that the mean incidences of MSS in four PD monkeys were higher in ipsilesional saccades than in contralesional saccades, in which the difference was statistically significant (*p* < 0.001, Figure 7C Wilcoxon signed-rank test) in monkey PK and HD who received bigger dose of MPTP injection. Such results are opposite to our expectation, i.e., the impairment of saccades should be more serious in contralesional direction than in ipsilesional direction. One possible explanation is that the dopaminergic system in basal ganglia over compensates with its function after being damaged in certain level, with the similar mechanisms after damaging of cerebral cortices (Dennis et al., 2010). Such results indicate that the incidence of MSS could serve as a complementary biomarker for the diagnosis of early PD even though typical motor symptoms are absent.

## Discussion

Although PD has been detected and studied for more than 200 years (Dorsey et al., 2018), objective and reliable biomarkers for the diagnosis of PD, particularly for its early diagnosis, are still lacking (Waninger et al., 2020). Saccadic eye movement might be a valuable behavioral biomarker because it reflects the physiological and cognitive functions of the brain and shows high test–retest constancy (Bargary et al., 2017). Indeed, previous studies have found that PD patients exhibit significant changes in certain saccadic parameters, such as the multiple-step pattern (Blekher et al., 2009) and vertical saccadic gain and error rate (Chehrehnegar et al., 2019; Irving and Lillakas, 2019; Waldthaler et al., 2019) in various oculomotor tasks. Researchers have argued that some changes in saccadic parameters could serve as complementary biomarkers for the diagnosis of PD (Chan et al., 2005; Armstrong, 2015; Patel et al., 2019). However, since most previous studies employed voluntary saccadic tasks, such as antisaccade and memory-guided saccade were usually difficult for elderly subjects to complete, particularly for patients with neurodegenerative diseases (Walker et al., 2000), the task difficulty highly restricted the application of measuring saccades in the diagnosis of PD. If the incidence of MSS in simply reactive saccades shows a significant difference between PD and control subjects, it might extend the application of MSS in the diagnosis of PD.

### The incidence of multiple step saccades in reactive saccades could serve as a complementary biomarker for the diagnosis of early Parkinson’s disease

In the present study, we applied a visually guided reactive saccade task, which is the easiest oculomotor task and only takes approximately 4 min to complete. The majority of participants completed the reactive saccadic task well (with average correct rates of 96.9, 93.3, 91.6, and 85.3% for the YHC, EHC, MCI, and PD groups, respectively). PD patients had significantly more MSS than YHC, EHC, and MCI participants (Figure 2). In addition, the incidence difference of MSS between vertical and horizontal saccades was significantly greater in PD patients than in YHC, EHC, and MCI participants (Figure 4). Such results indicate that the incidence of MSS in reactive saccades could serve as a complementary biomarker for the diagnosis of PD, with AUCs of 0.76 and 0.69 for discriminating PD from EHC and MCI, respectively.

Although the number of male and female participants is not perfectly balanced in the present study, it has been reported that there is no significant difference between male and female participants in performing visually guided saccade task (Bonnet et al., 2013). Thus, the unbalanced gender does not alter our results. Moreover, the age of MCI group was significantly older than PD group. Our results have shown that the incidence of MSS is significantly higher in EHC than in YHC (Figure 2), which indicates that aging would increase the occurrence of MSS. Nevertheless, the incidence of MSS in PD group is significantly higher than MCI group, which supports our argument that MSS could serve as a behavioral biomarker for the diagnosis of PD.

In addition, the correct rates of all monkeys but one (monkey PK: 74%) were >83%. The results of the monkey study show that the incidence of MSS was significantly higher in the three subclinical hemi-PD monkeys with MPTP injection doses ≥0.4 mg/kg than in healthy control monkeys (Figure 6). Such results indicate that the incidence of MSS could serve as a complementary biomarker for the diagnosis of early PD even though typical motor symptoms are absent.

### Discrimination between Parkinson’s disease and mild cognitive impairment indicating acceptable specificity by employing multiple step saccades to diagnose Parkinson’s disease

Since previous studies only compared the incidence of MSS between PD patients and EHC participants, the question regarding the specificity of MSS for the diagnosis of PD among other neurodegenerative diseases remains. It is important to evaluate the specificity of MSS for the diagnosis of PD because other neurodegenerative diseases also show abnormal saccadic behavior, e.g., MCI patients exhibit decreased saccadic latency, accuracy and velocity in reactive saccades (Chehrehnegar et al., 2021). Theoretically speaking, it is possible that the incidence of MSS might increase in MCI patients. Our results show that PD patients had significantly more MSS than MCI patients (Figure 2B), and we could discriminate PD from MCI with an AUC of 0.69 in the ROC analysis (Figure 5).

### Possible reasons for the significantly higher multiple step saccades incidence in vertical saccades than in horizontal saccades in Parkinson’s disease and mild cognitive impairment patients

For the first time, we showed that the incidence of MSS in vertical saccades was significantly higher than that in horizontal saccades in PD and MCI patients (Figure 4B). For PD patients, such results share certain commonalities with previous studies that reported a more severe impairment of saccades in the vertical direction (Rottach et al., 1996; Antoniades and Kennard, 2015; Lemos et al., 2016; Jung and Kim, 2019). An elongated saccadic latency (Lemos et al., 2016) and shortened saccadic amplitude (hypometria) in the vertical direction have been reported in PD patients (Jung and Kim, 2019). Here, we provide additional evidence to support the argument that the impairment of vertical saccades is more severe than that of horizontal saccades in PD. Since vertical and horizontal saccades are controlled by different brain regions and neural networks (Lemos et al., 2016; Takahashi and Shinoda, 2018; Irving and Lillakas, 2019), an intuitive thinking is that such directional differences in saccades might be due to the asymmetric impairment between these brain structures. Such an assumption is supported by a functional magnetic resonance imaging (fMRI) study, which shows that in PD patients, vertical reactive saccades cause higher activity in the right frontal eye field, cerebellar posterior lobe, and superior temporal gyrus than horizontal saccades (Lemos et al., 2016).

We are also, for the first time, able to report a higher incidence of MSS in vertical saccades than in horizontal saccades in MCI patients. The possible reason for this behavioral phenomenon might be the similar pathological alterations in MCI and PD patients, both of which are neurodegenerative diseases. Although there is no direct evidence to support this assumption, it is well known that MCI is the early stage of Alzheimer’s disease (AD) (Morris et al., 2015). According to the findings of previous studies, approximately 80% MCI patients develop to dementia in 6 years (Petersen et al., 2001). Moreover, PD and MCI are two common neurodegenerative diseases that share certain pathological changes such as alteration of neurotransmitter receptors and accumulation of misfolded proteins (Xu et al., 2012; Tosto et al., 2015).

### The possible neural mechanisms underlying the generation of multiple step saccades

Despite the advanced knowledge about the neural control of saccades, (Gaymard et al., 1998) there are few studies of the neuronal mechanisms underlying the generation of MSS. Thus, it is not clear how the brain develops MSS. Nonetheless, previous studies have found that some brain regions, i.e., the frontal cortex, basal ganglia and cerebellum, are involved in the generation of MSS (Avanzini et al., 1979; Kimmig et al., 2002; van Donkelaar et al., 2009). Stimulating the frontal eye field and supplementary eye field with transcranial magnetic stimulation (TMS) increased the incidence of MSS (van Donkelaar et al., 2009). Loss of dopaminergic neurons in the basal ganglia increased the incidence of MSS, as observed in PD patients (Kimmig et al., 2002; Blekher et al., 2009) and PD monkeys (Tereshchenko et al., 2015). Lesions in the cerebellum caused an increase in MSS incidence in human and non-human primates (Avanzini et al., 1979). Considering that the abovementioned brain regions have either direct or indirect (via the superior colliculus) anatomical connections with premotor circuits (comprised of omnipause and burst neurons) in the brainstem, (Munoz and Wurtz, 1995) the function of these regions in MSS generation is very likely to be a modulator rather than a generator. It is well known that the interinhibition between omnipause and burst neurons in premotor circuits causes pulse (saccade) and step (fixation) patterns of eye movements (Munoz and Wurtz, 1995). Moreover, a previous study found that electronically stimulating omnipause neurons during execution of a saccade immediately stopped the movement of the eyes and froze the eyes midway (Bergeron and Guitton, 2002). After releasing the electronic stimulation in omnipause neurons, the saccade resumed. Such a pattern of eye movement highly resembles MSS. Therefore, we assume that the lower oculomotor structures in premotor circuits of the brainstem are the generator of MSS, whereas the higher oculomotor structures in cortical and subcortical regions are the modulators of MSS.

### Limitations and future study

First, since the incidence of MSS has been studied only in one type of reactive saccade, i.e., the visually guided step saccade task (Figure 1A), it is interesting to study the incidence of MSS in other types of reactive saccades, such as the visually guided gap saccade task. Second, although the specificity of using MSS to diagnose PD is evaluated by comparing the incidence of MSS between PD and EHC and MCI, more studies are required to compare the incidence of MSS between PD and other neurodegenerative diseases, particularly movement disorder diseases such as essential tremor and Progressive Supranuclear Palsy. Third, PD patients were not in a full off-state in the present study and they were also combined with some cognitive deficits which might affect the comparison between PD and MCI patients. Fourth, although studies of subclinical hemi-PD monkeys directly explore the role of the basal ganglia in the generation of MSS, more studies are needed to systematically explore the neural mechanisms of MSS, such as single neuron recordings from oculomotor structures in the brain.

## Conclusion

Multiple step saccades in visually guided reactive saccades could be the biomarker for the early diagnosis of PD. In addition, the results from hemi-Parkinson monkeys indicate that the dopaminergic system in basal ganglia plays an important role in the development of MSS.

## Data availability statement

The raw data supporting the conclusions of this article will be made available by the authors, without undue reservation.

## Ethics statement

The studies involving human participants were reviewed and approved by Ethics Committee of Beijing Normal University and the Chinese PLA General Hospital (Medical School of Chinese PLA). The patients/participants provided their written informed consent to participate in this study. The animal study was reviewed and approved by Ethics Committee of Beijing Normal University.

## Author contributions

MZ and XX designed the experimental paradigm. WM, JW, ML, ZZ, and FJ performed the experiments. WM, JW, ZZ, HB, and MZ completed the MPTP injection operation of the four monkeys. WM analyzed the data. WM and MZ wrote and edited the manuscript. XX, ZL, and XL supervised the data collection and discussed the results. All authors contributed to the article and approved the submitted version.

## Conflict of interest

The authors declare that the research was conducted in the absence of any commercial or financial relationships that could be construed as a potential conflict of interest.

## Publisher’s note

All claims expressed in this article are solely those of the authors and do not necessarily represent those of their affiliated organizations, or those of the publisher, the editors and the reviewers. Any product that may be evaluated in this article, or claim that may be made by its manufacturer, is not guaranteed or endorsed by the publisher.
